# Associations between trajectories of social participation and functional ability among older adults: Results from the China Health and Retirement Longitudinal Study

**DOI:** 10.3389/fpubh.2022.1047105

**Published:** 2022-12-01

**Authors:** Jiaqin Xu, Jixiang Xu, Yingwei Chen, Yujie Wang, Guoyou Qin, Junling Gao

**Affiliations:** ^1^School of Public Health, Fudan University, Shanghai, China; ^2^Collaborative Innovation Cooperative Unit of National Clinical Research Center for Geriatric Diseases, Shanghai, China; ^3^Core Unit of Shanghai Clinical Research Center for Geriatric Diseases, Shanghai, China

**Keywords:** functional ability, social participation, healthy aging, trajectory, older adults

## Abstract

**Introduction:**

Functional ability (FA) and social participation (SP) are important indicators of healthy aging, both their trajectories are heterogeneous. It is little known about how the SP trajectories affects FA trajectories.

**Methods:**

FA was assessed by 20 items covering the ability of meeting basic needs and mobility. SP was assessed by frequency of participating in 10 social activities. Group-based trajectory modeling (GBTM) was used to identify the trajectories of FA and SP of the participants.

**Results:**

Two FA trajectories were identified: low baseline-decline tendency (16.1%) and high baseline-stable tendency (83.9%) trajectories. Two SP trajectories were also identified: low baseline-stable tendency (58.5%) and high baseline-increase tendency (41.5%) trajectories. After controlling for the potential covariates, participants among the high baseline-increase tendency SP trajectory group also had significantly higher odds ratios to be belonged in high baseline-stable tendency FA trajectory group (ORs = 2.64, 95%CI = 1.98–3.05).

**Conclusions:**

High-increasing social participation had a protective effect to maintain high baseline-stable tendency functional ability among older adults. These findings suggest social participation appears to have great benefits on promoting healthy aging in China.

## Introduction

The proportion of the world's population aged over 60 years old will dramatically increase by 22% in 2050 (1). Continuous global population aging poses huge challenges to health and social care systems. In order to address these challenges, the World Health Organization (WHO) proposed a public-health framework for healthy aging (2), which defined healthy aging as “the process of developing and maintaining the functional ability that enables wellbeing in older age” and emphasized that individuals' functional ability throughout the life-course was determined by their intrinsic capacity, environment, and the interaction of intrinsic capacity and environment. Functional ability enables people to be and to do what they have reason to value (3), which consists of people's abilities to meet basic needs; to learn, grow and make decisions; to be mobile; to build and maintain relationships and to contribute to society. One of the aims of the second WHO's action plan on aging and health termed “A Decade of Healthy Aging: From 2021 to 2030' is to improve older adults” intrinsic capacities and functional ability (FA) (4). Healthy aging is a dynamic process, so monitoring trajectories of healthy aging and its determinants is important for making public policies and interventions.

Older people are not a homogeneous group, researches have shown that heterogeneity of healthy aging exists in the intraindividual and interindividual (3, 5–9). Moreover, most existing studies mainly focused on population average trajectories of healthy aging and did not consider the existence of subgroups in the older population that may exhibit different trajectories than the majority of it (2, 10). Based on WHO healthy aging framework, FA is determined by the individuals' intrinsic capacity, their environment, and the interaction of intrinsic capacity with the environment (3). Researches have shown that demographic characteristics, socioeconomic status, health status, and behaviors predicated the trajectories of FA (11–13). Several studies also found that neighborhood physical environment (14) and social capital (15, 16) are associated with the trajectories of FA.

Social participation (SP) is an important indicator of successful/healthy aging (17–20), limited SP is a challenge to healthy aging in older age (21). Several longitudinal studies have found that high SP has protective effects for functional disability (22–24). Whereas, the majority of researches on social networks in later life emphasizes network decline and social isolation, an emerging trend stresses the bidirectional nature of network change (25). Researchers also found that social network growth was more common than social network shrinkage (26, 27). However, there was no study to assess the relationship between the trajectories of SP and the trajectories of functional ability.

The current study aims to assess trajectories of SP and trajectories of FA, and examine the former's protective effects to the latter using the data of the China Health and Retirement Longitudinal Study (CHARLS).

## Materials and methods

### Data source

This study used data of the CHARLS (http://charls.pku.edu.cn/), which is a nationally representative longitudinal survey to examine health and economic adjustments to the rapid aging of the population in China. A more detailed description was published elsewhere (28). In short, the national baseline survey (Wave 1) was conducted from June 2011 to March 2012 among 17, 708 respondents aged ≥45 years old who were recruited by multistage sampling strategy covering 28 provinces, 150 counties/districts, and 450 villages/urban communities across China. The CHARLS respondents are followed up every 2 years, using a face-to-face computer-assisted personal interview. Three follow-up visits were completed in 2013/2014 (Wave 2), 2015/2016 (Wave 3), and 2017/2018 (Wave 4). Ethical approval for collecting data on human subjects was received from the institutional review board at Peking University. Written informed consent was obtained from all the participants. In current study, inclusion criteria are: (1) respondents who aged ≥60 years old; (2) had completed all 4 waves surveys; (3) have no missing data of indicators of functional ability and social participation. Based on these inclusion criteria, 4,228 respondents were involved in this study (Supplementary Figure S1).

### Measures

#### Functional ability

Functional ability was outcome in this study based on International Classification of Functioning, Disability and Health (ICF) (29) and WHO's healthy aging framework (3). Referring to previous studies (30, 31), Functional ability assessment consists of ability of meeting basic needs and mobility in the current study. The ability of meeting basic needs was measured by self-reported difficulty in the following 6 domains of activities of daily living (ADL) and 5 domains of instrumental activities of daily living (IADL) (30): getting into or out of bed, bathing/showering, dressing, eating, using the toilet, controlling urination and defecation, doing household chores, shopping for groceries, preparing hot meals, managing money and taking medications. Mobility was measured by self-reported difficulty scale including the following 9 domains (31): running or jogging about 1 km, walking 1 km, walking 100 meters, getting up from a chair after sitting, climbing several flights of stairs without resting, stooping/kneeling/crouching, reaching/ extending arms above shoulder level, lifting or carrying weights over 5 kilograms, and picking up a small coin from a table. Twenty questions were given, and the response to each question was scored as 3 (No, I don't have any difficulty), 2 (I have difficulty but can still do it), 1 (Yes, I have difficulty and need help), or 0 (I cannot do it). The total functional ability score (ranged from 0 to 60) was obtained by summing the 20 questions' scores. A higher score indicated a higher level of functional ability (32). The Cronbach's alpha coefficient was 0.884 in this study.

#### Social participation

Respondents were asked to report the frequency they participated in 10 social activities (formal or informal) in the past month: (1) interacted with friends, (2) played ma-jong, chess, or cards or went to a community club, (3) provided help to family, friends or neighbors who do not live with you, (4) went to a sport, social or other kinds of club, (5) took part in a community-related organization, (6) did voluntary or charity work, (7) cared for a sick or disabled adult who does not live with you, (8) attended an educational or training course, (9) stock investment, (10) used the internet. The response to each social activity was scored as 0 (Non), 1 (Not regularly), 2 (Almost every week), or 3 (Almost daily). The total social participation score (ranged from 0 to 30) was obtained by summing the 10 social activities scores. A higher score indicated a higher level of social participation. This scale has been validated in a previous study (30). The Cronbach's alpha coefficient was 0.892 in this study.

#### Covariates

Based on the literature (11), covariates in this study included gender (male and female), age (5-year categories), marital status (married/cohabiting, divorced/separated, and widowed/never), education (illiteracy, no formal education, elementary school, middle school, and high school or above), self-rated health (very good, good, fair and poor), number of non-communicable chronic disease (NCD: 0, 1 and 2), smoking (never, ever and now) and drinking (none, less once a month and drinking).

#### Statistical analyses

To determine the best fitting discrete number and pattern of common functional ability and social participation trajectories, we used the group-based trajectory modeling (GBTM), which allows identifying unobserved groups of individuals following similar trajectories over age or time of a single outcome or behavior (33). The GBTM is a semi-parametric model for longitudinal data analysis (34). Compared to other models used for trajectory analysis, such as latent class analysis (35) and latent transition analysis (32), the model structure of GBTM is simpler because GBTM assumes that all individuals in the one trajectory group have the same behavior. As a result, the grouping results of GBTM may be easier to interpret and thus being a more practical choice for researchers (36, 37). Recently, the GBTM is increasingly being applied in healthy aging researches (9, 11, 38, 39). Some studies used the GBTM to find three (13, 39, 40) or four (11, 41) functional ability trajectories among older adults. Therefore, the GBTM is employed to discover the trajectory groups in our study.

All the participants in the current study participated 4 waves' survey every 2 years, we built trajectories by biennial intervals (year 0, 2, 4, 6 and 8). Hence, the mean elapsed time across waves was 2 years. The GBTM was conducted by following the next steps (42). Firstly, to find the optimal number of latent groups (trajectories) that best fit the data we performed several potential solutions with varying numbers of groups (from one to five) and orders of polynomials (linear or quadratic). After the number of groups was determined, the shape of each group's trajectory was estimated by specifying the order of the polynomial (linear, quadratic, or cubic). We consider the fitting indicators of Bayesian Information Criterion (BIC), the sample adjusted Bayesian Information Criterion (SABIC), the Akaike information criterion (AIC), and entropy (43, 44) to select the best model among the convergent models. The posterior probabilities for each individual being a member of the trajectories were calculated in the final model.

The baseline characteristics of the participants in different trajectory groups of functional ability were presented in counts, and the difference between groups were compared by Pearson's Chi-squared test. Multinomial logistic regression was employed to examine the association between SP trajectory and FA trajectory. Firstly, we examined the association between SP trajectory and FA trajectory without adjusting for any covariates. Next, we examined the association between SP trajectory and FA trajectory after controlling for potential covariates. We used R langue (45) [*lcmm* package *lcmm* (46)] and the Stata *traj* plugin (47) to conduct trajectory analysis. Considering the distributions of data, censored normal (cnorm) models were used for FA trajectory analysis, Zero-inflated Poisson (zip) models were used for SP trajectory analysis. All the other analyses were conducted with Stata 13.0 (48).

## Results

### Baseline characteristics

A total of 4,228 qualified older participants aged between 60 and 96 years with an average of 66.9 years (standard deviation 5.9 years) were included in our study. Among them, 49.2% were male, and 12.1% of them were aged ≥75 years old. Most (79.8%) people were married or cohabiting. More than one-third were illiterate. More than 60% of the older people self-reported “very good” or “good” health conditions; only 26.9% reported that they had no NCD. The prevalence of current smoking and drinking was 31.0 and 24.2%, respectively. Table 1 presented the detailed baseline characteristics of the study participants.

**Table 1:** Baseline sample characteristics and the sample by the different trajectory of functional ability.

	***N*** **(%)**	**Trajectory of functional ability**		***P***
		**L-D**	**H-M**	
**Gender**				*p* < 0.001
Male	2,078 (49.2)	217 (10.4)	1,861 (89.6)	
Female	2,150 (50.9)	465 (21.6)	1,685 (78.4)	
**Age (years)**				*p* < 0.001
60~	1,872 (44.3)	179 (9.6)	1,693 (90.4)	
65~	1,120 (26.5)	162 (14.5)	958 (85.5)	
70~	724 (17.1)	171 (23.6)	535 (76.4)	
75~	512 (12.1)	170 (33.2)	342 (66.8)	
**Marital status**				*p* < 0.001
Married/cohabiting	3,373 (79.8)	487 (14.4)	2,886 (85.6)	
Divorced/separated	149 (3.5)	19 (12.8)	130 (87.3)	
Widowed/never married	706 (16.7)	176 (24.9)	530 (75.1)	
**Education**				*p* < 0.001
Illiterate	1,478 (35.0)	371 (25.1)	1,107 (74.9)	
No formal education	888 (21.0)	132 (14.9)	756 (85.1)	
Elementary school	1,072 (25.4)	125 (11.7)	947 (88.3)	
Middle school	524 (12.4)	35 (6.7)	849 (93.3)	
High school and above	263 (6.2)	16 (6.1)	247 (93.9)	
**Self-rated health**				*p* < 0.001
Very good	863 (20.4)	46 (5.3)	817 (94.7)	
Good	2,067 (48.9)	237 (11.5)	1,830 (88.5)	
Fair	1,101 (26.1)	315 (28.6)	786 (71.4)	
Poor	195 (4.6)	83 (42.6)	112 (57.4)	
**Numbers of NCD**				*p* < 0.001
0	1,098 (26.9)	102 (9.3)	996 (90.7)	
1	1,212 (29.7)	157 (13.0)	1,055 (87.0)	
2	1776 (43.5)	396 (22.3)	1,380 (77.7)	
**Smoking**				*p* < 0.001
Never	2,482 (58.7)	460 (18.5)	2,022 (81.5)	
Ever	434 (10.3)	79 (18.2)	355 (81.8)	
Now	1,312 (31.0)	143 (10.9)	1,169 (89.1)	
**Drinking**				*p* < 0.001
None	2,927 (69.2)	545 (18.6)	2,382 (81.4)	
Less once a month	280 (6.6)	26 (9.3)	254 (90.7)	
Drinking	1,021 (24.2)	111 (10.9)	910 (89.1)	
**Social participation**				*p* < 0.001
L-D	2,475 (58.5)	530 (21.4)	1,945 (78.6)	
H-M	1,753 (41.5)	152 (8.7)	1,601 (91.3)	

L-D, Low baseline-decline tendency; H-M, High baseline-increase tendency; NCD, Non-communicable chronic disease.

### Trajectory types of FA score and SP score

**Figure 1:**
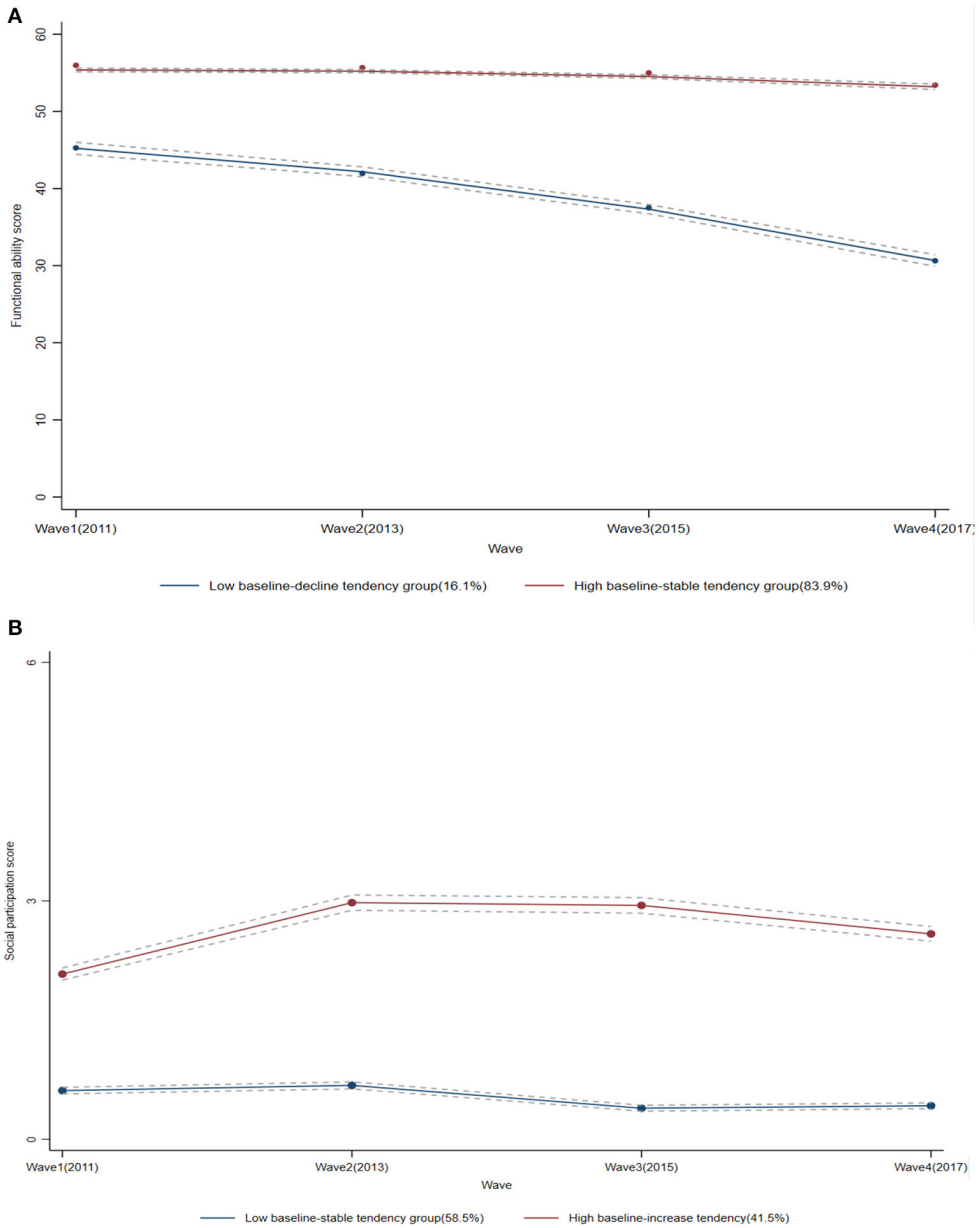
Trajectories of the functional ability scores and the social participation scores. The solid lines represent estimated values, and the dotted lines show the 95% CIs. **(A)** The functional ability scores; **(B)** the social participation scores.

With respect to trajectory analysis of FA score, the model of two trajectory groups with cubic algorithm among the 10 convergent models, had lower absolute AIC, BIC, SABIC, and highest entropy (Supplementary Table S1), which indicated two trajectories of FA score is optimal. In order to determine the best pattern of each trajectory of FA score, we ran 9 two-group models with varied algorithms (linear, quadratic, or cubic) for each trajectory, and found the two-group model with quadratic algorithm was the best model (Supplementary Table S2). To be more specific, as shown in Table 2 and Figure 1: (1) one group with a lower baseline FA score (intercept = 45.31) had a negative linear trajectory (*b* = −2.21, *P* < 0.001) and quadratic trajectory (*b* = −0.89, *P* < 0.001), indicating FA score was declined over the four waves (named low baseline-decline tendency group, accounting for 16.1% of total participants); (2) the other group with higher baseline FA score (intercept = 57.38) had a positive linear trajectory (*b* = 0.14, *P* > 0.05) and a negative quadratic trajectory (*b* = −0.39, *P* < 0.001), indicating FA score was stable over the four waves (named high baseline-stable tendency group, accounting for 83.9% of total participants).

**Table 2:** Maximum likelihood parameter estimates for trajectories functional ability and social participation (standard errors in parentheses).

	**Trajectories of functional ability**		**Trajectories of social participation**	
	**Low baseline-decline tendency**	**High baseline-stable tendency**	**Low baseline-stable tendency**	**High baseline-increase tendency**
Intercept	45.31 (0.33)^**^	57.38 (0.14)^**^	−0.49 (0.03)^**^	0.73 (0.02)^**^
Linear	−2.21 (0.49)^**^	0.14 (0.22)	0.85 (0.13)^**^	0.63 (0.06)^**^
Quadratic	−0.89 (0.16)^**^	−0.39 (0.07)^**^	−0.97 (0.13)^**^	−0.31 (0.05)^**^
Cubic			0.21 (0.03)^**^	0.04 (0.01)^**^
Group size (%)	16.1	83.9	58.5	41.5

** *p* < 0.001.

Following the above steps, we found the two-group model with cubic algorithm was best fitting trajectories of SP score (Supplementary Tables S3, S4). As shown in Table 2 and Figure 1, the trajectory of SP score for all older people was identified into 2 groups: (1) one group with lower baseline SP score (intercept = −0.49) had a positive linear trajectory (*b* = 0.85, *P* < 0.001) and cubic trajectory (*b* = 0.21, *P* < 0.001), but a negative quadratic trajectory (*b* = −0.97, *P* < 0.001), indicating SP score maintained stable over the four waves (named low baseline-stable tendency group, accounting for 58.5% of total participants); (2) the other group with higher baseline SP score (intercept = 0.73) had a positive linear trajectory (*b* = 0.63, *P* < 0.001) and cubic trajectory (b = 0.04, *P* < 0.001), but a negative quadratic trajectory (*b* = −0.31, *P* < 0.001), indicating SP score increased slightly over the four waves (named high baseline-increase tendency group, accounting for 41.5% of total participants).

### Association between trajectories of FA score and SP score

As showed in Table 3, multinomial logistic regression indicated that compared to the low baseline-stable tendency SP trajectory group, participants among the high baseline-increase tendency SP trajectory group had significantly higher odds ratios (ORs) to be belonged in high baseline-stable tendency FA trajectory group [(ORs = 2.87, 95% Confidence Interval (CI) = 2.37–3.48] without controlling for any covariate (Model 1). After controlling for potential covariates (Model 2), participants among the high baseline-increase tendency SP trajectory group remain had significantly higher ORs to be belonged in high baseline-stable tendency FA trajectory group (ORs = 2.64, 95%CI = 1.98–3.05).

**Table 3:** Multinomial logistic regression of association between trajectories of social participation and functional ability.

	**Model 1 (95%CI)**	**Model 2 (95%CI)**
**Gender**		
Male		Ref.
Female		0.54 (0.41–0.70)
**Age (year)**		
< 50		Ref.
50~		0.64 (0.50–0.82)
60~		0.35 (0.27–0.45)
70~		0.23 (0.17–0.31)
**Marital status**		
Married/cohabiting		Ref.
Divorced/separated		1.20 (0.69–2.11)
Widowed/never married		0.78 (0.61–0.99)
**Education**		
Illiterate		Ref.
No formal education		1.28 (0.99–1.65)
Elementary school		1.43 (1.10–1.85)
Middle school		2.21 (1.48–3.30)
High school and above		2.37 (1.34–4.19)
**Self-rated health**		
Very good		Ref.
Good		0.45 (0.32–0.65)
Fair		0.18 (0.12–0.25)
Poor		0.10 (0.06–0.16)
**Numbers of NCD**		
0		Ref.
1		0.86 (0.64–1.15)
2		0.54 (0.42–0.71)
**Smoking**		
Never		Ref.
Ever		0.63 (0.44–0.89)
Now		0.97 (0.74–1.28)
**Drinking**		
None		Ref.
Less once a month		1.31 (0.79–2.17)
Drinking		0.95 (0.73–1.23)
**Social participation**		
Low baseline-stable tendency	Ref.	Ref.
High baseline-increase tendency	2.87 (2.37–3.48)	2.46 (1.98–3.05)

NCD, Non-communicable chronic disease. Model 1 was conducted without adjusting for any covariates.

## Discussion

Previous studies indicated that functional ability was a predictor of quality of life (49), wellbeing (50), and mortality (51, 52) among older adults. Optimizing functional ability is one of the goals of the decade of healthy aging (2021–2030) (3) the United Nations proposed. Monitoring trajectories of functional ability and their determinants have the potential to identify when and how to intervene at different life stages to maximize the chance of healthy aging for the population and susceptible subgroups. The current study identified two distinct trajectories of functional ability among Chinese older adults aged >60 years during a 7-year follow-up period. One group was featured with a low baseline and decline tendency functional ability trajectory, another group was featured with a high baseline and stable tendency functional ability trajectory. However, in a study from the Philippines, the functional ability trajectories of middle-aged and older Filipino women were identified into 4 categories, named robust, late onset, early onset, and recovery (11). A study of Taiwanese middle-aged and older adults aged 50 years and older identified participants' functional ability trajectories into 3 categories, named early, mid, and late onset (39). A Dutch study identified the functional ability trajectories of 645 middle-aged and older adults aged 55–85 years in the Amsterdam area into 3 categories, named successful, late decline, and early decline (13). In two studies from Japan, one study identified the functional ability trajectories of Japanese older people aged 65 years and over into 3 categories, named slowly declining, persistently disabled, rapidly declining (40). Another study identified that of Japanese older people aged 60 years and over into 3 categories, named minimal disability, late-onset disability, early-onset disability, and moderate disability (41). The reasons for the differences in trajectory categories across studies may be multifaceted, such as the age composition of participants, differences in functional ability measurement scales, local cultural and lifestyle differences. Besides confirming those were female, poorer SRH, higher age, and less educated were susceptible to be low baseline-decline tendency group, we also found multimorbidity was a predictor of low baseline-decline tendency functional ability.

Regarding the focus of this study, we found that 58.5% of participants maintained their social participation at low baseline and remained stable level, while 41.5% of them held high baseline and increasing social participation during a 7-year period. These findings demonstrated that the mean trajectory of social participation of older adults tended to deteriorate slowly over time, but varied change trajectories existed, consistent with previous studies (53, 54). Although the existed studies examined the association between social participation and functional ability (23, 30, 55), there were few studies to examine the effect of social participation trajectories to functional ability. A recent study found that high- and moderate-stable social activity may slow the rate of cognitive decline (56). In the current study, we found that older adults with high baseline-increase tendency social participation had significantly higher ORs of high baseline-stable tendency functional ability (ORs = 2.64, 95%CI = 1.98–3.05) compared with the low baseline-stable tendency SP trajectory group. Therefore, it is beneficial to maintain functional ability to promote social participation among older adults.

There are several explanations for association between social participation and functional ability decline. Firstly, social participation may increase the availability of social support and social cohesion (19), thereby, buffering stress and increasing psychological resilience (57, 58). Studies have demonstrated that higher level of psychological resilience may protect against increases in functional limitations (59, 60) in later life. Secondly, social participation may promote healthier behaviors, such as adequate fruit/vegetable intake (61), increasing physical activity and reducing sedentary time (62), and better sleep quality (63). Thirdly, social participation may exert direct physiological benefits, such as preserving hippocampal function (64) and lowering inflammatory markers (65, 66).

Our study has some limitations that should be mentioned. Firstly, the observed relationship between SP and FA could not be interpreted as the causative association limited by the nature of the observational study design. Randomized controlled studies are needed to confirm their causation. Secondly, the results may be biased by the exclusion of older adults with missing data of functional ability. One of the reasons for older adults couldn't finish the questionnaire might be functional limitations, so the functional ability may be overestimated in the current study. Thirdly, functional and social participation were assessed by the widely used self-reported questionnaires, which were not evaluated for reliability and validity. Moreover, we adjusted many covariates in the analyses, but residual confounders still may influence the results, such as attributes of neighborhood where the participants lived. However, these covariates were not available in the CHARLS database. Despite the limitations, the study has some strengths. First, it is a longitudinal study with large national sample data for a long period. Second, we assessed varied dimensions of social participation (i.e., social connections, informal social participation and volunteering) (19) by asking the participants participating the frequency of each social activity.

## Conclusion

In conclusion, we identified two distinct trajectories of functional ability as well as social participation respectively among community-dwelling older adults during a 7-year observation period. More importantly, we found the significant association between high baseline-increase tendency social participation and high baseline-stable tendency functional ability among older adults. These findings suggest social participation appears to have great benefits on promoting healthy aging in China.

## Data availability statement

Publicly available datasets were analyzed in this study. This data can be found at: The datasets used to analyze and supported the findings of this study are available on the website of the China Health and Retirement Longitudinal Study (CHARLS) at http://charls.pku.edu.cn/index/en.html. To access and use this survey data for research purpose, an approval should be obtained from the CHARLS team at Peking University.

## Ethics statement

The studies involving human participants were reviewed and approved by Institutional Review Board at Peking University. The patients/participants provided their written informed consent to participate in this study.

## Author contributions

JiaX and JixX contributed to perform the data analyses and wrote the manuscript. YC and YW contributed to the data cleaning and preparation of manuscript. GQ and JG contributed to the design and conception of the study and help perform the analysis with constructive discussions. All authors had full access to all data in the study and had final responsibility for the decision to submit for publication.

## Funding

This work was supported by the National Key Research and Development Program of China (Grant Numbers 2018YFC2002000 and 2018YFC2002001) and the National Natural Science Foundation of China (Grant Number 82173634). The funder had no role in study design, data collection, data analysis, data interpretation, or writing of the report.

## Conflict of interest

The authors declare that the research was conducted in the absence of any commercial or financial relationships that could be construed as a potential conflict of interest.

## Publisher's note

All claims expressed in this article are solely those of the authors and do not necessarily represent those of their affiliated organizations, or those of the publisher, the editors and the reviewers. Any product that may be evaluated in this article, or claim that may be made by its manufacturer, is not guaranteed or endorsed by the publisher.
