# Supplementary material for: Associations between trajectories of social participation and functional ability among older adults: Results from the China Health and Retirement Longitudinal Study
Source: Front Public Health. 2022 Dec 1;10:1047105. doi: 10.3389/fpubh.2022.1047105 (PMC9751478; doi:10.3389/fpubh.2022.1047105)
Supplement: Supplementary file 1 [file Data_Sheet_1.docx]

**Supplemental file**

**2011**: 17,708

Died: 370, lost: 737

**2013**: 6,358

Died: 444, lost:778

Died: 644, lost:876

**2015**: 5,873

**2018**：5131

Excluded because of missing data: 903

Excluded as age <60: 10,243

**Figure S1 Flowchart of participants from 2011-2018 in CHARLS**

| **Model** (number of trajectories and algorithm) | **Convergent** | **AIC** | **BIC** | **SABIC** | **Entropy** |
| --- | --- | --- | --- | --- | --- |
| one group-linear | yes | 114284.4 | 114322.5 | 114303.4 | 1 |
| one group-quadratic | yes | 113895.7 | 113959.2 | 113927.4 | 1 |
| one group-cubic | yes | 113805.1 | 113900.4 | 113852.7 | 1 |
| two group-linear | yes | 112882.7 | 112939.9 | 112911.3 | 0.917 |
| two group-quadratic | yes | 113903.7 | 113992.6 | 113948.1 | 0.001 |
| two group-cubic | yes | 112281.9 | 112408.9 | 112345.3 | 0.921 |
| three group-linear | yes | 112888.7 | 112964.9 | 112926.8 | 0.45 |
| three group-quadratic | yes | 112384.8 | 112499.1 | 112441.9 | 0.395 |
| three group-cubic | no | 112291.9 | 112450.6 | 112371.2 | 0.524 |
| four group-linear | yes | 112894.7 | 112989.9 | 112942.3 | 0.297 |
| four group-quadratic | yes | 112392.8 | 112532.5 | 112462.6 | 0.359 |
| four group-cubic | no | 112317.7 | 112508.2 | 112412.9 | 0.276 |
| five group-linear | no | 112900.7 | 113015 | 112957.8 | 0.318 |
| five group-quadratic | no | 112400.8 | 112565.9 | 112483.3 | 0.358 |
| five group-cubic | no | 112323.1 | 112545.3 | 112434.1 | 0.226 |

**Table S1 Fitting indices of varied trajectories of functional ability**

**Table S2 Fitting indices of varied trajectory patterns of functional ability**

| Algorithms of each trajectory | BIC | SABIC | AIC | entropy |
| --- | --- | --- | --- | --- |
| Linear/linear | 51871.87 | 51867.71 | -51848.66 | 0.916 |
| Linear/quadratic | 51858.82 | 51853.97 | -51831.75 | 0.916 |
| Linear/cubic | 51863.26 | 51857.71 | -51832.31 | 0.916 |
| Quadratic/linear | 51859.2 | 51854.35 | -51832.12 | 0.915 |
| **Quadratic/quadratic** | **51848.05** | **51842.51** | **-51817.11** | **0.916** |
| Quadratic/cubic | 51852.55 | 51846.31 | -51817.74 | 0.916 |
| Cubic/linear | 51863.53 | 51857.98 | -51832.58 | 0.915 |
| Cubic/quadratic | 51852.46 | 51846.22 | -51817.65 | 0.916 |
| Cubic/cubic | 51856.98 | 51850.05 | -51818.3 | 0.916 |

**Table S3 Fitting indices of varied trajectories of social participation**

| **Model** (number of trajectories and algorithm) | **Convergent** | **AIC** | **BIC** | **SABIC** | **Entropy** |
| --- | --- | --- | --- | --- | --- |
| one group-linear | yes | 69090.7 | 69128.8 | 69109.72 | 1 |
| one group-quadratic | yes | 68795.2 | 68858.7 | 68826.96 | 1 |
| one group-cubic | yes | 68745.0 | 68840.2 | 68792.55 | 1 |
| two group-linear | yes | 69096.7 | 69153.8 | 69125.23 | 0 |
| two group-quadratic | no | 68803.2 | 68892.1 | 68847.65 | 0 |
| two group-cubic | yes | 67516.6 | 67643.6 | 67580.06 | 0.931 |
| three group-linear | yes | 68043.8 | 68119.9 | 68081.82 | 0.556 |
| three group-quadratic | yes | 67714.6 | 67828.9 | 67771.66 | 0.376 |
| three group-cubic | no | 67526.6 | 67685.4 | 67605.93 | 0.678 |
| four group-linear | yes | 68049.8 | 68145 | 68097.33 | 0.266 |
| four group-quadratic | yes | 67722.6 | 67862.3 | 67792.35 | 0.264 |
| four group-cubic | no | 67536.6 | 67727.1 | 67631.8 | 0.31 |
| five group-linear | no | 68055.8 | 68170 | 68112.85 | 0.232 |
| five group-quadratic | no | 67730.6 | 67895.7 | 67813.03 | 0.287 |
| five group-cubic | no | 67546.6 | 67768.9 | 67657.64 | 0.26 |

**Table S4 Fitting indices of varied trajectory patterns of social participation**

| Algorithms of each trajectory | BIC | SABIC | AIC | entropy |
| --- | --- | --- | --- | --- |
| Linear/linear | 28867.21 | 28863.74 | -28847.87 | 0.804 |
| Linear/quadratic | 28745.09 | 28740.94 | -28721.89 | 0.807 |
| Linear/cubic | 28732.57 | 28727.72 | -28705.49 | 0.807 |
| Quadratic/linear | 28854.05 | 28849.89 | -28830.84 | 0.803 |
| Quadratic/quadratic | 28841.05 | 28836.19 | -28813.97 | 0.809 |
| Quadratic/cubic | 28735.48 | 28729.93 | -28704.54 | 0.806 |
| Cubic/linear | 28824.75 | 28819.9 | -28797.67 | 0.806 |
| Cubic/quadratic | 28713.06 | 28707.51 | -28682.11 | 0.809 |
| Cubic/cubic | **28711.09** | **28704.85** | **-28676.28** | **0.808** |

^a^ BIC: Bayesian Information Criterion

^b^ SABIC: Sample Adjusted Bayesian Information Criterion

^c^ AIC: Akaike information criterion
